# Curbing Inflammation through Endogenous Pathways: Focus on Melanocortin Peptides

**DOI:** 10.1155/2013/985815

**Published:** 2013-05-07

**Authors:** Tazeen J. Ahmed, Trinidad Montero-Melendez, Mauro Perretti, Costantino Pitzalis

**Affiliations:** ^1^William Harvey Research Institute, Barts and The London School of Medicine, Charterhouse Square, London EC1M 6BQ, UK; ^2^The Centre for Experimental Medicine & Rheumatology, William Harvey Research Institute, Barts and The London School of Medicine & Dentistry, 2nd Floor, John Vane Science Centre, Charterhouse Square, London EC1M 6BQ, UK

## Abstract

The resolution of inflammation is now known to be an active process, armed with a multitude of mediators both lipid and protein in nature. Melanocortins are peptides endowed with considerable promise with their proresolution and anti-inflammatory effects in preclinical models of inflammatory disease, with tissue protective effects. These peptides and their targets are appealing because they can be seen as a natural way of inducing these effects as they harness endogenous pathways of control. Whereas most of the information generated about these mediators derives from several acute models of inflammation (such as zymosan induced peritonitis), there is some indication that these mediators may inhibit chronic inflammation by modulating cytokines, chemokines, and leukocyte apoptosis. In addition, proresolving mediators and their mimics have often been tested alongside therapeutic protocols, hence have been tested in settings more relevant to real life clinical scenarios. We provide here an overview on some of these mediators with a focus on melanocortin peptides and receptors, proposing that they may unveil new opportunities for innovative treatments of inflammatory arthritis.

## 1. Inflammation: Onset and Resolution

**Figure 1:**
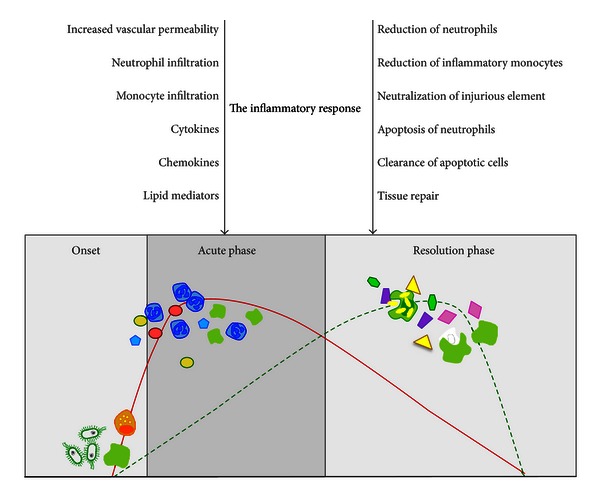
The inflammatory response. Stimuli such as tissue injury or microbial invasion trigger the release of chemical mediators (complement, cytokines, eicosanoids, and other autacoids) that activate the leukocyte recruitment (onset). Neutrophils are the first cell type to be recruited, and then peripheral blood monocytes also accumulate at the inflammatory site (acute phase). These monocytes will eventually differentiate into a more phagocytic phenotype helping to neutralize the injurious element and to clear the tissues off apoptotic neutrophils (resolution phase). This proresolving macrophage (and the involvement of stromal cells cannot be excluded here either) orchestrate resolution, by releasing and/or responding to proresolving mediators, some of which have been discussed in this review (see main text). Eventually, fully differentiated cells that have cleared the site by debris, dead cells, and bacteria will leave (via the lymphatic?) and the previously inflamed tissue or organ will regain its functionality, with return to homeostasis.

One novel approach to the area of inflammation, developed over the last twenty years, is the concept of resolution of inflammation. Current therapies suppress active processes of inflammation, for example, NSAIDs (nonsteroidal anti-inflammatory drugs) block cyclo-oxygenases, glucocorticoids inhibit generation of multiple cytokines, and biologics such as anti-TNF*α* and anti-CD20 therapies, target specific effectors or antigens. However, this may be only half the story. The story of inflammation begins with a tissue insult originating from an infection, trauma, or damage. The affected tissue secretes signals including autacoids, plasma-derived mediators such as kinins and complement factors, culminating with the now prominent cytokines and chemokines. There are multiple molecules that constitute a distress signal. This leads to an initial recruitment of neutrophils, (or eosinophils, upon parasite attack) which mop up any initial infection and call in macrophages, which are also inflammatory. Once neutrophils and macrophages have cleared the inflammation, the neutrophils undergo apoptosis, the macrophage changes its phenotype into a proresolving and tissue repair one, and then leaves and the tissue should return to its baseline uninflamed state [1]. However this return to baseline is not, as was once thought, characterised solely by absence of the inflammatory insult but it results also from a positive process with its own armamentarium of mediators that bring the tissue from an inflammatory state back into its normal resting state (Figure 1).

There are several processes of clearance of inflammation that lead to the return to the normal state (catabasis) [2]. Exclusion of the primary insult, for example, phagocytosis of invading bacteria, is foremost as this stops the synthesis of proinflammatory mediators. There is then the breakdown of the proinflammatory stimuli and also the cessation of production of these proinflammatory cytokines, chemokines, and other inflammatory mediators such as MMPs (matrix metalloproteinases) and proteolytic enzymes. This is the process that is targeted by most current therapy. Then there is the removal of the inflammatory cell infiltrate. This can be local cell death, usually by apoptosis followed by phagocytosis by macrophages (M2 phenotype, with anti-inflammatory remit) that then leave the site by lymphatic drainage [3]. Some of these macrophages themselves may die by apoptosis and be cleared by other resident cells. The crucial concept is that ingestion of the apoptotic neutrophils by macrophages (efferocytosis) would prevent the appearance of necrotic cells, which eventually will release their harmful content, therefore perpetuating the inflammatory response. In addition, this process is nonphlogistic; that is, it does not induce an inflammatory response [4]. Some cells might recirculate systemically and leave the site of inflammation [5]. The resolution phase of an acute inflammatory process can be defined in histological terms as the interval from maximum neutrophilic infiltration to the absence of neutrophilia [1].

There is now a host of mediators that are involved in the resolution phase of inflammation. Some of these are autacoids like adenosine, locally generated hormones like melanocortins and somatostatin, bioactive lipids like lipoxins, resolvins, protectins and maresins, proteins like heme oxygenase 1, annexin A1, galectins, and erythropoietin. Due to space limitation, we will discuss here a few examples of proresolving proteins and peptides in the melanocortin system.

## 2. The Melanocortin System as Archetypal for Proresolving Endogenous Mediators and Targets

In 1950, Philip Hench won the Nobel prize for treating patients with rheumatoid arthritis (RA) with cortisone [6, 7]. What is less well known is that he treated 6 patients with adrenocorticotrophic hormone (ACTH) with equally good results, as reported in that seminal paper. ACTH is the prototype of the melanocortins and its anti-inflammatory actions have been confirmed and formed the basis for its use in the clinical management of inflammatory arthritides such as in the treatment of gout, where it is still used in the USA today. A placebo controlled trial of synacthen, a synthetic form of ACTH, in patients with RA showed an additional benefit which lasted three months after two injections on alternate days [8]. ACTH was evaluated in the treatment of gout patients, with relative contraindications to NSAIDS, ACTH was found to have good effect over and above that which would be expected from the release of endogenous cortisol alone [9]. The later discovery of the proopiomelanocortin system with a number of melanocortins and melanocortin receptors (MCR) has improved our understanding of the biological basis of these effects.

### 2.1. Melanocortin Receptors

The melanocortin system (Table 1) encompasses five melanocortin receptors, their ligands (agonists and antagonists), and the accessory proteins. The melanocortin receptors are a family of five small stimulatory G protein-coupled receptors, termed MC_1_ to MC_5_, initially identified as neuropeptide receptors in mice and humans in the early 1990s [21–27]. Each receptor has seven transmembrane domains, with an extracellular amino-terminus and short cytosolic carboxy-terminus. The melanocortin 2 receptor (MC_2_) is the only one of the five which has been shown to require an accessory protein for translocation to the cell membrane [28]. However the presence of the accessory proteins (MRAP1 or MRAP2 (melanocortin receptor accessory protein)) may have an effect on the surface expression of the other melanocortin receptors and their ability to be activated by agonists [29, 30].

All melanocortin receptors signal via the cyclic AMP (cAMP) pathway, activating adenylate cyclase resulting in increased intracellular cyclic AMP [31–33]. Activation of certain MCRs has also been shown to mobilise calcium from intracellular stores [34–36] in certain cell types or conditions. For example, activation of MC_3_ with alpha-MSH can result in increases in intracellular calcium in the presence of the protein kinase A inhibitor H-89 [31]. Similarly, if the cAMP pathway is blocked then MC_1_ can signal via intracellular calcium mobilisation or the inositol trisphosphate pathway [37, 38]. MC_1_ has been shown to affect the NF*κ*B pathway by protecting I*κ*B*α* from degradation leading to a downregulation of inflammatory cytokines and chemokines [33, 39].

In terms of distribution, melanocortin receptors are found in the brain and in peripheral tissues. It is notable that MC_1_, MC_3_, and MC_5_ are expressed on multiple cells of the immune system suggesting a role in inflammation. Of note to the rheumatologist, MC_1_ and MC_5_ are present in human articular chondrocytes [40] and rheumatoid synovial fibroblasts. These cells are known to be part of the chronic immune response of rheumatoid arthritis and represent a source of effector cells for endogenous ligand.

### 2.2. Melanocortin Receptor Ligands

The ligands for the melanocortin receptors are derived from the proopiomelanocortin system. Proopiomelanocortin (POMC) is the precursor protein, from which prohormone convertases cleave the melanocortin stimulating hormones (MSH) alpha-, beta- and gamma-MSH and ACTH as well as the nonmelanocortin peptides, beta-lipotropin, gamma-lipotropin, and beta-endorphin. Initially, POMC and its related components were thought to be restricted to the pituitary but now have been shown to have a wider distribution [41].  Table 2 summarizes the endogenous and synthetic melanocortin peptides.

MC_2_ only responds to ACTH while the other melanocortin receptors respond to the melanocortin stimulating hormones to differing degrees [42]. MC_1_ responds to alpha-MSH>ACTH>gamma-MSH, as does MC_5_, whilst MC_3_ responds to gamma-MSH=ACTH>alpha-MSH. As well as endogenous agonists, there are endogenous antagonists in both the mouse and human system [43]. These are known as agouti and agouti-related protein in the mouse and agouti signalling protein in the human. Other regulators in mice are mahogany, syndecan and the mahogunin ring finger 1 [44].

### 2.3. Anti-Inflammatory and Antiarthritic Actions of Melanocortin Peptides

The anti-inflammatory actions of alpha-MSH have been shown using *in vitro* studies on stable cell lines and human primary cells, as well as *in vivo* models of diseases such as rheumatoid arthritis, colitis or ischaemia reperfusion injury. Alpha-MSH was initially found to be an antipyretic, able to counteract the pyrogenic activities of IL6 and TNF*α* [45]. Manna and Aggarwal then showed that alpha-MSH suppressed proinflammatory cytokine production by monocytes in response to bacterial lipopolysaccharide, by inhibiting NF*κ*B translocation to the nucleus [33]. Not only does alpha-MSH suppress proinflammatory cytokines, but also it can activate the production of anti-inflammatory cytokines such as IL10 from monocytes [46] and keratinocytes [47]. Alpha-MSH has been shown to be inhibitory in several inflammatory models. It is effective in experimental contact dermatitis and suppresses the sensitisation and elicitation phase of the immune response. Alpha-MSH induces hapten specific tolerance when given intravenously and this response is dependent on the induction of IL10 [48]. This finding has been taken forward in the nickel-induced contact eczema model in humans where a topical application of alpha-MSH reduced disease [49]. Alpha-MSH has been used in a model of cutaneous vasculitis induced by LPS and the peptide was able to reduce vascular damage and haemorrhage by downregulating cell adhesion molecules crucial for the extravasation of leukocytes to the site of inflammation [50]. Alpha-MSH has been topically applied to an airways model of allergy sensitised to ovalbumin, proallergic cytokines were reduced, and the anti-inflammatory action of alpha-MSH was dependent on IL10 [51].

Melanocortin agonists have been investigated in models of stroke encompassing mouse, rat, and gerbil models and also global and local ischaemic models. Gerbils, given ten minutes of global cerebral ischaemia by the occlusion of both carotid arteries, had reduced neuronal death, hippocampal damage and improved functional recovery if treated with an alpha-MSH derivative with a longer half-life (Nle^4^, D-Phe^7^ alpha-MSH, NDP-MSH) between three and nine hours after insult. Interestingly MC_4_ blockade abrogated the effects of the NDP-MSH suggesting the activity of MC4R in this process [52]. In human studies, alpha-MSH levels in the plasma have been used as a biomarker for predicting functional recovery from stroke [53].

Alpha-MSH and its analogues have also been used in preclinical models of renal and lung injury, secondary to sepsis or other forms of injury. It has been shown in multiple models to ameliorate injury with improvements in histology and plasma creatinine compared to controls. AP214, a nonselective melanocortin agonist derived from alpha-MSH, has been used in a sepsis-induced kidney injury model. Treatment with AP214 was delayed until six hours after the onset of sepsis and still reduced damage to the kidney as monitored by histological score, tubular damage, and serum creatinine; these effects were associated also with an improved liver function [16]. AP214 also reduced serum TNF*α* and IL10 and showed evidence of reduced NF*κ*B activation. There was also an improvement in survival rate in both lethal sepsis groups (improved from 0% survival to 10% survival) and sublethal sepsis groups (an improvement from 40% survival to 70% survival). This has been reflected in other studies of kidney injury models where alpha-MSH was given up to 6 hours after injury, observing increased recovery and protection against renal injury [54].

Alpha-MSH also ameliorates liver inflammation—as assessed following endotoxin induced inflammation in mice- even if given 30 minutes after onset, with decreased neutrophils infiltration and also decreased gene expression of chemotactic cytokines such as MCP1 (monocyte chemoattractant protein) and IL8 as well as TNF*α* [55]. Severe tissue injury in the lung can lead to acute respiratory distress syndrome as can renal ischaemic reperfusion injury with similar pathways activated in both organs. Alpha-MSH can inhibit lung oedema decrease injury score and leukocyte infiltrate as well as decreasing serum creatinine and improving histology score in the kidney. Gene expression of TNF*α* and ICAM1 (intercellular adhesion molecule) is reduced in the lung after treatment with alpha-MSH. Alpha-MSH also prevented the degradation of I*κ*B, phosphorylation of p38 mitogen activated protein kinase and decreased AP1 binding suggesting that alpha-MSH can operate through various pathways to modulate the inflammatory response, rather than just triggering one method of dampening inflammation [56].

Melanocortin agonists have been used in the treatment of various experimental arthritis models. AP214 is a peptide pan-agonist which has been shown to reduce the disease score in a mouse model of arthritis and induce proresolving properties (increased phagocytosis) in macrophages [17]. Carrier technology has been applied to alpha- and gamma-MSH and used in the CIA (collagen induced arthritis) and urate peritonitis models showing effective amelioration of inflammatory parameters of the two experimental diseases [11]. This approach aims to facilitate the targeting to inflammatory sites of unstable peptides such as melanocortins, by fusion with the latency-associated peptide (LAP) of TGF*β*1 through a cleavable matrix metalloproteinase linker [57]. 

Alpha-MSH has been used to treat adjuvant arthritis in rats with an increase in body weight, reduction of the arthritis score, and erosions [58]. POMC gene therapy has been used to treat adjuvant arthritis in rats with a reduction in paw swelling after adjuvant injection as well as thermal hypersensitivity [59]. Melanocortins have also been studied in models of gouty arthritis. Alpha-MSH and a small peptide derivative (CKPV-2) have been shown to inhibit the ability of monocytes to produce neutrophils chemoattractants and activating compounds in response to urate crystals [60]. The melanocortin peptide ACTH_4–10_ has been shown to reduce neutrophil accumulation in an *in vivo* model of crystal-induced peritonitis and to inhibit *in vitro* macrophage activation with reduced chemokine KC release [61]. By using the mixed MC_3_/MC_4_ antagonist SHU9119, this study identified MC_3_ as being responsible for these actions, since peritoneal macrophages do not express MC_4_. Also the agonist melanotan II, a stable pan-agonist at all receptors, gave similar results as the alpha-MSH derivative [61, 62]. In the same system, two MC_3_ agonists MT-II and gamma-MSH also inhibited neutrophil accumulation and release of cytokine and chemokines from macrophage. Furthermore MC_3_ is expressed in the C57BL6 mouse and Sprague Dawley rat peritoneal macrophages, as determined by Western blot. ACTH reduced joint size and inhibited neutrophils accumulation in rat knee joints injected with urate crystals. SHU9119 abrogated the effectiveness of ACTH in this model while gamma-MSH showed similar protective qualities [63]. Further evidence suggesting that MC_3_ is important in this model came with the efficacy of nonselective and selective MC_3_ agonists in the amelioration of urate crystal-induced peritonitis in a mouse colony bearing a nonfunctional MC_1_. This was further supported by presence of MC_3_ protein in mouse peritoneal macrophages [64]. [D-Trp^8^]gamma-MSH (a gamma-MSH derivative with preference for activating mouse MC_3_) afforded protection when used for the treatment of rat gout arthritis or urate peritonitis, and but not when used in MC_3_ deficient mice (only the urate peritonitis model has been tested), again suggesting a role for MC_3_ in controlling the inflammation produced by urate crystals [65]. The same compound has been shown to be efficacious in murine peritonitis despite a nonfunctional MC_1_, again guiding us to believe that MC_3_ might be more relevant in this mouse model of gout [66]. Overall these experiments show the efficacy of ACTH and its derivatives, natural and synthetic, in the treatment of mouse and rat models of gout and suggest that MC_3_ is the receptor mediating these effects.

### 2.4. Melanocortins in Human Arthritis

Little is known about the effects of melanocortins on human arthritis other than the effects of ACTH in rheumatoid arthritis and gout which have been known about since the 1950s [6, 7, 67–72]. The clinical efficacy of ACTH in gouty arthritis was reevaluated in the 1990s [9] and this retrospective study confirmed an efficacy over and beyond what one would expect from the release of endogenous cortisol. In the United States, ACTH is part of the clinical armamentarium for gout, especially for the treatment of patients with contraindications to NSAIDs. Catania et al. discovered elevated levels of alpha-MSH in the synovial fluid of rheumatoid arthritis and juvenile chronic arthritis patients compared to those with osteoarthritis (OA). Using paired samples, these authors also showed that the levels of alpha-MSH were elevated in synovial fluid as compared to serum. The concentrations of alpha-MSH were proportional to the degree of inflammation [73]. Bohm's and Grassel's groups have reported presence of melanocortin receptors 1 and 5 in human chondrocytes and have proposed a role for the melanocortins in the osteoarticular system [74]. Yoon et al. showed a reduction in MMP13 production and p38 kinase phosphorylation when human chondrosarcoma cells were pretreated with alpha-MSH and then stimulated with TNF*α*. This was independent of ERK and JNK kinases but reliant on p38 kinases and NF*κ*B [75]. Addition of alpha-MSH to TNF*α*-activated human chondrocytes reduced production of proinflammatory cytokines and increased the release of the anti-inflammatory cytokine IL10 [76].

### 2.5. Would New Therapeutics Emerge from Research on Melanocortins?

We conclude this overview by highlighting the therapeutic potential that the area of the resolution of inflammation may retain. For space limitation, we focus on the melanocortin research only, though it is clear that other effectors of resolution and their targets would also be endowed with promising opportunities. Only the future will tell if new therapeutics will indeed be developed out of this research effort.

Given the data from preclinical models and the success of alpha-MSH and its derivatives as well as *de novo* agonists, melanocortin ligands have been taken forward into clinical trials for further investigation in humans. The minimum peptide sequence from alpha-MSH that can activate MC receptors is a tri-peptide (KPV). However, although active, it has a very short half-life and much work has been based on modifying alpha-MSH and its derivatives to extend their duration of action. There is much effort on melanocortin peptides, with the aim of producing a preparation, that is, easy to deliver, specific to its target tissue, more selective, and with a longer half-life. 

Possible side effects of melanocortin receptor stimulating drugs are skin pigmentation and increased risk of melanoma due to activation of MC_1_, hypertension, and behavioural disturbances due to activation of MC_3_, MC_4_, and MC_5_. Pan-agonists may activate the yawning and stretching reflexes stimulated by MC_4_. An important aspect to consider for small molecules targeting MC_3_ would be their inability to cross the blood brain barrier, thus preventing unwanted actions on food intake and central control of blood pressure [77].

Despite these potential side effects, melanocortin-based therapeutics are generally safe and well tolerated by patients. In addition, ACTH (which activates all melanocortin receptors, as discussed above) is currently used for the treatment of gout, but it displays efficacy also for proteinuric nephropathies [78], exacerbations of multiple sclerosis [79], and several rheumatic disorders [8, 9, 72, 80], indicating that targeting the melanocortin system might be a genuinely valid therapeutic approach. However, the paucity on appropriate randomized, controlled, double-blind trials to evaluate the efficacy of this drug has limited its use as well as development of melanocortin-based therapies. 

The perception may be changing right now. In fact, we are experiencing a renaissance of the melanocortin field as many drugs are (or have been) subjected to clinical trials to treat a variety of conditions (http://clinicaltrials.gov). For example, the drug RM-493, a selective MC_4_ peptide agonist from Rhythm Pharmaceuticals, Inc. is currently on a phase-II trial to evaluate its antiobesity effects. The drug bremelanotide (Palatin Technologies), which activates MC_1_ and MC_4_ receptors, is currently under investigation for female sexual arousal disorder, although the effects on blood pressure have questioned the risk/benefit ratio of bremelanotide for this indication. Action Pharma A/S completed a phase-II clinical trial using the compound AP214 (described earlier in this review) to assess the prevention of kidney injury in patients undergoing cardiac surgery. In addition, the FDA Office of Orphan Products Development is running a trial to assess the effects of alpha-MSH on acute renal failure. A third study (phase II, completed) focused on nephropathies is conducted by Radboud University, in which they aimed to evaluate the effects of a synthetic ACTH on idiopathic membranous nephropathy. Diabetic nephropathy is another indication under evaluation in a phase IV clinical trial conducted by Southeast Renal Research Institute. ACTH is also being evaluated for the treatment of systemic lupus erythematosus and multiple sclerosis (Questcor Pharmaceuticals, Inc., phase IV and I, resp.). The peptide afamelanotide, an alpha-MSH derivative with increased stability and potency developed by Clinuvel Pharmaceuticals Limited, is currently under investigation for the treatment of erythropoietic protoporphyria (phase-III, recruiting), solar urticaria (phase II, completed), vitiligo (phase I), and for the treatment of acne vulgaris due to its anti-inflammatory properties (phase II, completed). Of note, companies such as MSH Pharma, Inc. include in their pipeline melanocortin drugs to treat conditions such as rheumatoid arthritis and inflammatory bowel disease, highlighting that there is a renewed current interest in developing melanocortin-based therapies for chronic inflammatory diseases and that melanocortin drugs are ready for translation.

## Figures and Tables

**Table 1:** The melanocortin system.

	Melanocortin Receptors				
	MC_1_	MC_2_	MC_3_	MC_4_	MC_5_
Endogenous agonists	*α*MSH=ACTH>*β*MSH>*γ*MSH	ACTH	*γ*MSH>ACTH=*α*MSH=*β*MSH	*α*MSH=ACTH>*β*MSH>*γ*MSH	*α*MSH>ACTH=*β*MSH≫*γ*MSH

Distribution	Skin Melanocytes Keratinocytes Endothelial cells Mucosal cells Adipocytes Chondrocytes Osteoblasts Macrophages Monocytes Dendritic cells Mast cells Neutrophils CD8+ T cells B Lymphocytes	Adrenal glands Adipocyte Osteoblasts Dendritic cell Chondrocyte	Hypothalamus Macrophages Monocytes Dendritic cells CD4+ T cells B lymphocytes	Hypothalamus Dendritic cells Osteoblasts	Exocrine glands Sebocytes Macrophages Dendritic cells Mast cells Chondrocyte CD4 T cells B lymphocytes NK cells

Signalling pathways	cAMP ERK1/ERK2	cAMP	cAMP Intracellular [Ca^2+^]	cAMP	cAMP Intracellular [Ca^2+^] Jak/STAT

Biological functions	Skin pigmentation Inflammation Wound healing	Steroidogenesis	Energy homeostasis Inflammation	Energy homeostasis Food intake Erectile function	Exocrine glands function, Inflammation Defensive behaviour

Role in disease/Potential use	Skin cancer Inflammation Alopecia areata (?) Vitiligo	Familial glucocorticoid deficiency	Inflammation Gouty arthritis Obesity Tuberculosis (?)	Obesity Cachexia Sexual dysfunction	Seborrheic dermatitis Acne vulgaris Inflammation

**Table 2:** State of the art for the development of melanocortin agonists.

Compound	Classification	Activity	Effects	References
*α*MSH	Endogenous	Pan agonist	Anti-inflammatory Skin pigmentation	[10]
*β*MSH	Endogenous	Pan agonist		
*γ*MSH	Endogenous	Pan agonist with increased MC_3_ selectivity	Anti-inflammatory	[11]
Agouti related peptide	Endogenous	Antagonist, MC_3_, MC_4_	Skin pigmentation	
Agouti signalling protein	Endogenous	Antagonist, MC_1_, MC_3_, MC_4_	Skin pigmentation	
D-Trp^8^-*γ*MSH	Synthetic peptide	Agonist with increased MC_3_ selectivity	Anti-inflammatory (arthritis)	[12]
NDP-*α*MSH (MT-I)	Synthetic peptide	Pan agonist	Anti-inflammatory	[10]
MT-II	Synthetic peptide	Pan agonist	Anti-inflammatory	[13]
KPV	Synthetic peptide	MC_1_ agonist	Anti-inflammatory	[10]
KPT	Synthetic peptide	Pan agonist	Anti-inflammatory	[10]
(CKPV)2	Synthetic peptide	Pan agonist	Anti-inflammatory	[14]
GKPV	Synthetic peptide on beads	Pan agonist	Anti-inflammatory (melanoma)	[15]
AP214	Synthetic peptide	Pan agonist	Anti-inflammatory (sepsis and arthritis)	Action Pharma A/S [16, 17]
HP228	Synthetic peptide	Pan agonist	Protective in acute models of inflammation and organ damage	[18]
BMS470539	Small molecule	Agonist MC_1_	Inhibits LPS response	[19]
ME10501	Small molecule	High affinity mMC_1_, hMC_4_	Neuroprotective	[20]
Bremelanotide	Small molecule	Agonist MC_1_ and MC_4_	Prevents organ dysfunction	Palatin Technologies
SHU-9119	Synthetic peptide	Antagonist at MC_3_ and MC_4_, agonist for MC_1_ and MC_5_	Experimental tool	
Afamelanotide	Synthetic peptide	Pan agonist	Vitiligo, acne vulgaris, erythropoietic protoporphyria, solar urticaria	Clinuvel Pharmaceuticals
RM-493	Synthetic peptide	Agonist MC_4_	Obesity	Rhythm Pharmaceuticals, Inc
Czen001, 002	Synthetic peptide	Agonist	Anti-infective Anti-inflammatory	MSH Pharma
